# Supersaturated Oxygen Delivery & Acute MI: Current Evidence, Practical Issues, and Future Research

**DOI:** 10.2174/011573403X351088250802160032

**Published:** 2025-08-05

**Authors:** Adhvithi Pingili, Mounika Reddy Vadiyala, Maneeth Mylavarapu, Muhammad Bilal, Narayana Varalakshmi Akula, Mallareddy Banala, Neelima Katukuri

**Affiliations:** 1 Department of Internal Medicine, MedStar Union Memorial Hospital, Baltimore, MD 21218, USA;; 2 Department of Internal Medicine, Maimonides Medical Center, Brooklyn, NY 11219, USA;; 3 Department of Public Health, Adelphi University, NY 11530, USA;; 4 Department of Internal Medicine, Detroit Medical Center, Detroit, MI, 48201, USA;; 5 Department of Internal Medicine, Carle Foundation Hospital, IL 61801, USA;; 6 Department of Internal Medicine, Division of Cardiovascular Medicine, University of Miami Miller School of Medicine, Miami, FL 33136, USA;; 7 Department of Cardiology, University of Central Florida, Orlando, FL 32816, USA

**Keywords:** Supersaturated oxygen, animal models, myocardial infarction, acute myocardial infarction with hyperoxemic therapy, improved echocardiographic regional wall motion score index, ST-elevated myocardial infarction

## Abstract

**Introduction:**

Acute myocardial infarction is a significant global health issue, with a high mortality rate. Early intervention, specifically coronary angiography, has been shown to improve patient outcomes significantly. Recent technological advances have introduced novel therapeutic interventions like Supersaturated Oxygen (SSO_2_) Therapy, which promises enhanced recovery post-ischemia. To evaluate the efficacy and potential benefits of SSO_2_ therapy in improving post-ischemic outcomes, including left ventricular function, myocardial scarring, and overall cardiac morphology.

**Methods:**

This review synthesizes findings from early clinical trials and pre-clinical animal studies focusing on the application of SSO_2_. The therapy involves delivering oxygen at ten times the normal level directly to the infarcted artery to facilitate high oxygen diffusion before restoring downstream flow.

**Results and Discussion:**

Pre-clinical studies demonstrate that SSO_2_ therapy enhances Left ventricular ejection fraction (LVEF), increases mean arterial PaO_2_, reduces myocardial apoptosis, and decreases myocardial scarring and infarct sizes. Clinical trials, including the Acute Myocardial Infarction with Hyperoxemic Therapy (AMIHOT I and AMIHOT II), have shown improvements in echocardiographic regional wall motion and have helped in preventing myocardial dilatation and remodeling.

**Conclusion:**

SSO_2_ therapy presents a promising advance in the treatment of myocardial infarction. While early results are favorable, indicating significant improvements in cardiac function and tissue preservation, long-term follow-up studies are necessary to determine the impact on mortality rates, recurrence of ischemic events, and healthcare resource utilization.

## 1 INTRODUCTION

Acute myocardial infarction is one of the leading causes of mortality and morbidity worldwide. During the acute phase, cardiogenic shock and ventricular arrhythmias are major drivers in mortality, contributing to almost 10% of in-hospital mortality [1]. However, during the initial pre-hospital and in-hospital phase, mortality due to ST-elevated myocardial infarction (STEMI) remains unaltered by intense lipid-lowering, antithrombotic, and anti-inflammatory therapies [2-8]. Regarding prevalence, Nader *et al*., reported the global prevalence of Myocardial infarction in people above 60 years was 9.5% [9]. With the rising global prevalence and unaltered mortality rates, extensive clinical trials have consistently demonstrated that early coronary angiography is associated with significantly improved outcomes for patients facing ischemic heart events. This outcome enhancement can be attributed to the fundamental principle that revascularization substantially improves myocardial oxygen delivery and recovery.

Ischemia and reperfusion represent the opposing yet interconnected pathophysiological processes in coronary artery disease. Reperfusion injury is a paradoxical response with mechanisms including but not limited to the generation of Reactive oxygen species, lipid peroxidation, and endothelial activation leading to the release of pro-inflammatory cytokines [10-13]. Recent developments in medical technology have opened doors to further optimizing post-ischemic event care, including interventions like Supersaturated Oxygen (SSO_2_), which provides about ten times the level of oxygen when applied to the infarcted artery that allowing for high oxygen diffusion prior to restoration of flow downstream. The improved oxygen delivery upstream with super-saturated oxygen is seen to help prevent cellular edema in animal models [10]. The Acute Myocardial Infarction with HyperOxemic Therapy II (AMIHOT II) trial [14] and Evaluation of intracoronary hyperoxemic oxygen therapy in acute anterior myocardial infarction (IC-HOT study) [7] both demonstrated a reduction in infarct size and an early safety profile that led to its approval by the Food and Drug Administration (FDA). This exciting approach holds the promise of further improving patient outcomes after ischemic events, but its full clinical implications and effectiveness are still subjects of ongoing research and evaluation.

This review explores the growing body of research on SSO_2_ delivery, hyperoxemic blood flow, and microvascular improvements as potential strategies for enhancing outcomes in patients following ischemic cardiac events. Our review aims to offer a comprehensive understanding of the currently available literature, pitfalls, and future courses by delving into the available literature.

## 2 METHODS

A comprehensive search was conducted in prominent databases (PubMed, Web of Science, Google Scholar, and Scopus) to identify and include studies on SSO_2_ and Acute Myocardial Infarction (AMI). Additionally, ongoing studies and research registered on ClinicalTrials.gov were included to capture the latest advancements in the field. Screening was done to identify studies based on their abstracts, and full-text articles were obtained for detailed evaluation. Both observational and experimental studies (animal studies, cohort studies, and randomized controlled trials) were included. Exclusion of the studies was done if they were not relevant to the review or were not published in English.

## 3 REVIEW

### 3.1 Pre-clinical Animal Studies

Preclinical studies have laid the foundation for clinical trials of SSO_2_ therapy (previously referred to as aqueous oxygen). The SSO_2_ therapy, a catheter-based technique designed to increase the partial pressure of oxygen in coronary arterial blood to very high levels (760-1,000 mm Hg), aims to increase oxygen delivery to endothelial cells and myocardial tissue *via* diffusion through plasma, was developed by Dr. J. Richard Spears *et al*. to address myocardial salvage in STEMI [15-17]. By improving oxygenation, the hypothesis was that myocardial cells on the verge of dying could be salvaged, and endothelial swelling could be reversed to relieve microvascular obstruction (MVO), further improving microvascular blood flow and oxygen delivery. As a catheter-based method for delivering regional arterial hyperoxemia, it provides a potentially more practical solution for critically ill patients by enabling precise control of oxygen levels in specific regional arteries. Fig. (**1**) outlines the possible mechanism of action of SSO_2_.

Spears *et al*. initially demonstrated the feasibility of aqueous oxygen therapy in canine models by showing that intra-arterial aqueous oxygen infusion in dogs subjected to coronary hypoxemia preserved Left ventricular ejection fraction (LVEF), in contrast to the decline in LVEF in dogs with low-flow hypoxemic and normoxemic perfusion [18]. Later, Kantor *et al*. conducted a study using the porcine model to assess the efficacy of SSO_2_ therapy. They observed that SSO_2_ infusion (PaO_2_ of 622 mm Hg) resulted in enhanced LV function and reduced infarct size, particularly in pigs with lower baseline LVEF (<50%), compared to normoxemic auto perfusion [19]. Subsequent preclinical studies have explored the benefits of intracoronary SSO_2_ infusion for acutely enhancing reperfusion after myocardial infarction (MI) [16, 17, 20-22].

Spears *et al*. conducted a study in a canine model where a 90-minute coronary artery occlusion was induced, followed by a 30-minute normoxemic auto-reperfusion and then a 90-minute SSO_2_ therapy, while the control groups received normoxemic reperfusion using a roller pump [16]. The introduction of intracoronary SSO_2_ therapy post auto-reperfusion in the test group led to increased mean arterial PaO_2_ (530 mm Hg), significant enhancement in LVEF, improvement in Left Ventricular (LV) regional wall motion abnormalities and ST-segment deviation, and reduction of premature ventricular beats, compared to the normoxemic auto-reperfusion group. Additionally, there was a marked increase in regional myocardial blood flow (0.92 ml/g/min *vs.* 0.43 ml/g/min) in the previously ischemic zone of the SSO_2_ group compared to the control group (*P* < 0.01). This study underscored that SSO_2_ improves LV function, electrocardiographic parameters, and regional myocardial blood flow during reperfusion and that inadequate reperfusion occurs at the microvascular level despite patency of the epicardial infarct-related coronary artery.

In a separate study by Spears *et al*. on pigs, it was demonstrated that a 60-minute balloon occlusion in the Left Anterior Descending (LAD) artery, followed by 15 minutes of normoxemic auto-perfusion, then 90 minutes of intracoronary hyperoxemic perfusion (mean PaO_2_ of 834 mm Hg), resulted in enhanced LVEF (62% at 105 minutes and 60% at 165 minutes *vs.* 45% and 48% at 105 minutes and 45% and 43% at 165 minutes) and smaller myocardial infarct size (<20% of the LV *vs.* 70%-80% of the LV) compared to the control groups, that included continuous normoxemic auto-perfusion and normoxemic reperfusion [17]. The study also emphasized better preservation of myocardial structure in the hyperoxemic group, which was associated with lower hemorrhage score and myeloperoxidase levels, the latter indicating reduced oxygen-free radical damage. These findings highlighted the potential of SSO_2_ in reducing infarct size and improving LV function.

Furthermore, SSO_2_ therapy demonstrated a marked reduction in myocardial apoptosis, signifying its potential to curtail cell death in affected myocardial areas. Myocardial ultrastructure analysis revealed less endothelial cell edema and preservation of endothelial nuclei in the SSO_2_ group compared to controls. These results were consistent across various studies, suggesting SSO_2_ therapy improves functional outcomes and positively impacts myocardial structural integrity [20, 23, 24].

In a study involving swine, Spears *et al*. also demonstrated that even when SSO_2_ therapy was administered 24 hours after reperfusion onset following a one-hour occlusion of the LAD, there were significant improvements in infarct size and LVEF compared to the control group. These findings highlight the sustained vulnerability to treatment of myocardial stunning due to uncorrected microvascular hypoxia, which is potentially reversible with SSO_2_ therapy [25].

Another study by Kaluza *et al*., involving a 60-minute coronary artery occlusion in pigs, assessed the potential adverse effects of SSO_2_ post-primary Percutaneous Coronary Intervention (PCI) with stent implantation [26]. Kaluza *et al*.'s study found that 90 minutes of SSO_2_ infusion (PaO_2_ 760-1,000 mm Hg) post-primary PCI and stent implantation did not lead to coronary thrombi or adverse effects on coronary arteries or myocardium, compared to normoxemic controls. While histological analysis noted a trend towards reduced myocardial scarring in the SSO_2_ group, echocardiography confirmed improvement in LV function. This study confirmed the safety and potential efficacy of SSO_2_ therapy in acute myocardial infarction settings [26].

Kaluza *et al*. further investigated the chronic myocardial, coronary, and systemic effects of intracoronary SSO_2_ therapy on swine with normal and ischemic-reperfused myocardium [26]. Forty swine were stented in the left anterior descending coronary artery and randomized to receive either a 90-minute infusion of SSO_2_ or normoxemic saline. The study found no evidence of coronary thrombosis or any myocardial or systemic toxicity from SSO_2_ therapy. Additionally, there was a trend towards reduced microscopic myocardial scarring, reduced infarct size, and improved global and regional contractility in the SSO_2_ group, indicating potential benefits in heart recovery post-MI. This study, in contrast to previous studies, showed the absence of SSO_2_ therapy-related adverse effects on the stented coronary arteries as well, along with normal and non-infarcted myocardium [27].

Several early animal studies indicated that hyperoxemic reperfusion using aqueous oxygen was not only safe but also effective in addressing regional hypoxemia. These studies showed that it raised oxygen partial pressures without leading to bubble formation and contributed to improved left ventricular functioning, with the potential to reduce the size of the infarct. The TherOx system has been specifically designed for intracoronary infusion of blood enriched with aqueous oxygen, aiming to enhance oxygen delivery to the myocardium [23]. Although the idea of administering large amounts of oxygen does not increase reactive oxygen species, but helps in reducing them [17]. Table **1** outlines the key findings of the pre-clinical animal studies.

### 3.2 Early Clinical Trials

Preclinical research validated the safety and efficacy of SSO_2_, leading to the initiation of a series of clinical trials in patients with STEMI. The Dixon *et al*. phase 1 feasibility study was a multicenter clinical trial that involved 29 patients with acute STEMI who were reperfused with primary PCI and then administered hyperoxemic oxygen using the TherOx^®^ device for 60 to 90 minutes [28]. All patients underwent successful hyperoxemic reperfusion without any therapy-related adverse events. The study observed an improvement in the echocardiographic regional wall motion score index and an increase in mean LVEF from 48.6% immediately after angioplasty to 51.8% at 24 hours. This improvement in LVEF continued progressively over the following months, reaching 54.4% at 1 month and 56% at 3 months, thus concluding that SSO_2_ therapy was safe, effective, and well-tolerated [28].

The AMIHOT, a prospective and multicenter trial including 269 patients suffering from acute anterior or large inferior STEMI, all enrolled within 24 hours of symptom onset from 23 sites in three countries [29]. Following successful primary PCI, the patients were randomized to receive either a 90-minute intracoronary SSO_2_ therapy or standard auto reperfusion. The SSO_2_ therapy was delivered through an intracoronary infusion catheter positioned in the proximal segment of the infarct-related artery, lasting for 90 minutes. Infarct size was assessed using Tc 99m sestamibi Single photon emission computed tomography (SPECT) imaging at 14-21 days post-procedure, while the change in echocardiographic regional wall motion score index in the infarct area was evaluated at three months post-infarction.

Although no significant differences were observed in infarct size or regional wall motion score index between the two groups, SSO_2_ therapy did show promising results in a subgroup of 105 patients with anterior infarcts who were reperfused within 6 hours of symptom onset. In these patients, SSO_2_ therapy was associated with a smaller infarct size (9% *vs.* 23% of the LV; *P* = 0.04) and a better regional wall motion score (−0.75 *vs.* −0.54; *P* = 0.03) when compared to the control group. Additionally, SSO_2_ therapy led to a decreased area under the ST-segment deviation time curve over 3 hours, indicating superior reperfusion stability compared to the control group. However, the two groups had no significant differences in major adverse cardiac events [29].

The AMIHOT I trial included a sub-study by Warda *et al*. in which myocardial contrast echocardiography was performed at 24 hours and one month [30]. Warda *et al*. also reported a significant increase in the LV end-diastolic and end-systolic volumes in the control group, wherein the volumes remained unchanged in the SSO_2_ group. Additionally, LVEF increased significantly in the SSO_2_ group 49% to 54%; *P* <0.05) but remained unchanged in the control group (51% to 50%). The study showed that SSO_2_ therapy administered to patients with acute anterior MI within 6 hours of symptom onset effectively prevented adverse LV remodeling, including LV dilatation, and led to an improvement in LV ejection fraction. Since adverse post-infarction LV remodeling is linked to worse early and late clinical outcomes in myocardial infarction [31-33], SSO_2_ could be a significant adjunct therapy [30].

The investigators of AMIHOT I concluded that intracoronary hyperoxemic reperfusion with SSO_2_ therapy was safe and well-tolerated. Although the therapy did not demonstrate a reduction in infarct size, improvement in regional wall motion abnormalities, or stabilization of ST-segment resolution on the electrocardiogram in the overall patient population, the trial helped identify a subset of anterior STEMI patients who were reperfused within 6 hours as potential beneficiaries of the therapy. These findings served as the rationale for the subsequent AMIHOT II trial [14].

The AMIHOT II, a prospective, randomized trial, was conducted with 301 patients with anterior STEMI treated with PCI within 6 hours of symptom onset and had thrombolysis in myocardial infarction (TIMI) grade 2-3 flow after the procedure [14]. Eligible patients were then randomized (2.8:1) to a 90-minute infusion of SSO_2_ or control normoxemic auto-reperfusion. The study utilized a Bayesian hierarchical modeling approach to analyze the primary endpoints, incorporating data from the AMIHOT-I trial and weighting the data based on the degree of similarity between the two trials. In the pooled study, the treatment group experienced a statistically significantly smaller median infarct size (IS) than the control group (18.5% *versus* 25% of LV mass), as measured by single photon emission computed tomography at two weeks. In patients with a baseline LV ejection fraction <40%, the treatment group experienced even greater myocardial salvage, with an absolute IS of 33.5% in controls and 23.5% in patients treated with SSO_2_.

Regarding clinical outcomes such as major advanced cardiac events, SSO_2_ was non-inferior to the control group. However, the study was underpowered to show a significant reduction in IS at 14 days, but it was positive when pooling the positive subgroup from the AMIHOT-I cohort. The study concluded that SSO_2_ therapy, administered post-PCI for 90 minutes, safely reduced infarct size in patients with anterior STEMI who received successful PCI within six hours of symptom onset. Although some safety concerns emerged, such as an increased risk of access site-related events, primarily hematomas in the SSO_2_ group (due to the larger 7–8 Fr femoral access), and an increase in thrombotic events (concerns regarding the intra-coronary positioning of a catheter for SSO_2_ delivery), these findings were not statistically significant [14].

Following the AMIHOT II trial, a pilot study was conducted to evaluate the safety and feasibility of delivering hyperoxemic blood through a standard 5F diagnostic catheter positioned in the left main coronary artery (LM) instead of using an infusion catheter in the left anterior descending artery (LAD) [34]. Additionally, the infusion time was reduced from 90 to 60 minutes by increasing the flow rate from 75 mL/min to 100 mL/ min to ensure the same dose of SSO_2_ solution. The study enrolled 20 patients with acute anterior STEMI who presented within 6 hours of symptom onset. The primary safety endpoint, target vessel failure, occurred in only one patient (5%) within 30 days, which was due to stent thrombosis with reinfarction, and no hemorrhagic complications were reported.

The median infarct size was observed to be 13.7% at 3-5 days and reduced to 9.6% at 30 days. Additionally, the mean myocardial salvage index at 3-5 days was 50%, as assessed by cardiac magnetic resonance (CMR). The study concluded that delivering SSO_2_ therapy for 60 minutes through a 5F diagnostic catheter in the LM was both feasible and safe, and it resulted in a relatively small infarct size in patients with anterior STEMI who had undergone successful primary PCI [34].

The IC HOT study was developed and conducted under the guidance of the FDA, following the insights gained from the pilot study [15]. This study demonstrated the safety and feasibility of infusing oxygenated blood into the left main coronary artery over 60 minutes using a standard 5F catheter. In this prospective, open-label, single-arm trial, 100 patients with anterior STEMI who presented within 6 hours of symptom onset received a 60-minute infusion of SSO_2_ therapy through a 5F diagnostic catheter in the left main artery, following reperfusion and stenting in the proximal or mid-LAD.

The primary safety endpoint was net adverse clinical events (NACE), a composite measure including reinfarction, target vessel revascularization (TVR), stent thrombosis, severe heart failure (HF), or TIMI major/minor bleeding. The study reported an infarct size of 24.1% at four days, which decreased to 19.4% at 30 days, as determined by cardiac Magnetic Resonance Imaging (MRI). These findings were comparable to the average infarct size of approximately 20% observed in the SSO_2_ treatment group of the AMIHOT II trial at 14 days [14].

The 30-day NACE rate at 30 days was 7.1%, which was notably lower than the expected 10.7% based on the control population of 112 subjects from the INFusion of intracoronary Abciximab and Aspiration Thrombectomy in Patients with Anterior ST-segment Elevation Myocardial Infarction (INFUSE-AMI) INFUSE-AMI study, which had similar inclusion and exclusion criteria. This was also lower than the 13.1% NACE rate observed in the AMIHOT II study, confirming the safety of the protocol [35]. Importantly, no deaths were reported, and only one case each of stent thrombosis and severe HF was observed. Additionally, four patients experienced TIMI minor bleeding, with no incidence of severe bleeding reported.

One of the key findings of the Evaluation of Intracoronary Hyperoxemic Oxygen Therapy in Acute Anterior Myocardial Infarction (IC-HOT) study was that SSO_2_ therapy appeared to prevent the progression of LV dilatation and remodeling, which is typically seen in the month following STEMI. This was evidenced by the reduction in the LV end-systolic volumes measured over 30 days (−8.1% at 30 days). This finding confirms the results of a prior substudy by Warda *et al*. that evaluated LV recovery from AMIHOT I (−11.0% at 30 days) [30]. The investigators concluded that the optimized infusion protocol of SSO_2_ therapy after successful primary PCI for acute anterior STEMI was feasible and had a favorable safety profile with a low rate of adverse events at 30 days.

Finally, a propensity-matched study compared 1-year outcomes from IC-HOT to those of a control group from the INFUSE-AMI study [36]. The results revealed that patients treated with SSO_2_ had a lower incidence of cardiovascular mortality (0% *vs.* 4%, *P*=0.04) and new-onset HF or hospitalizations for HF (0% *versus* 7.4%, *P*=0.01) compared to the control group. There were no discernible differences between the groups in rates of reinfarction or TVR. Notably, only one patient who received SSO_2_ experienced stent thrombosis within one year, as opposed to four control patients (1.2% *versus* 4.9%, respectively; *P*= 0.17).

In light of these outcomes, the Food and Drug Administration (FDA) approved SSO_2_ therapy on April 2, 2019, and it is now indicated for non-shock left anterior descending coronary artery (LAD) STEMI treated successfully with PCI within 6 hours of symptom onset. SSO_2_ is the pioneering FDA-approved device-based therapy for AMI cardio protection [37].

Furthermore, in 2021, the Incorporating Supersaturated Oxygen in Shock (ISO-SHOCK trial, NCT04876040) was initiated, with an expected completion date in 2025 [38]. This multi-center, randomized-controlled (1:1) pilot study is designed to assess the safety and feasibility of administering SSO_2_ therapy into the coronary arteries of patients suffering from acute myocardial infarction and cardiogenic shock (STEMI-CS) following successful reperfusion *via* PCI within six hours of symptom onset.

Additionally, a larger trial, AMIHOT III (NCT0474 3245), is currently underway. This multi-center, randomized, post-approval study aims to evaluate the delivery of hyperoxemic SSO_2_ therapy for 60 minutes in patients with anterior MI who have undergone successful reperfusion *via* PCI within 60 minutes of symptom onset. The study, which is actively enrolling 434 participants, will assess the rate of net adverse clinical events over 12 months, with an estimated completion date in 2026 [39]. Table **2** outlines the key findings of the early clinical trials.

### 3.3 Pitfalls

Although SSO_2_ therapy has the potential of being a groundbreaking approach in the management of AMI, there are significant pitfalls that need to be considered carefully prior to optimizing its clinical utility. Despite the promising preclinical and early clinical trials and subsequent approval by the FDA, the evidence supporting the safety and efficacy of SSO_2_ remains limited because of a lack of prospective studies with extended follow-up periods. Furthermore, there is limited evidence on the appropriate patient population, which causes struggle among cardiologists to determine which AMI patients will benefit from SSO2 therapy, ultimately leading to variability in treatment decisions and potentially suboptimal outcomes.

The delivery of SSO_2_ treatment involves complex procedural techniques, including catheter navigation, device deployment, and optimization of oxygen delivery parameters. These procedures require greater technical expertise. This can be challenging, particularly in healthcare settings with limited interventional expertise or resources. The studies reviewed have shown inconsistent outcome measurement and reporting, such as infarct size, LVEF, and adverse effects. These variations could potentially be due to varying imaging modalities like SPECT *vs.* CMR, differences in time points for assessing infarct size and LVEF. Consistent and standardized outcome measurements are essential in the effective evaluation of SSO_2_ therapy.

Implementation of SSO_2_ therapy can be associated with significant upfront costs related to equipment procurement, staff training, and procedural infrastructure development, along with ongoing costs associated with device maintenance, disposable supplies, and quality assurance measures. These costs may pose a financial burden for institutions, especially in the resource-limited setting.

Patient safety is also another notable concern in the current understanding of SSO_2_ therapy. Intracoronary positioning of SSO_2_ delivery catheter poses a significant risk of thrombotic events. Furthermore, limited data exist on the long-term safety implications of SSO_2_ therapy. Reimbursement for interventional cardiology procedures is based on the availability of clinical evidence demonstrating their safety, efficacy, and potential clinical benefits compared to existing standards of care. Generating high-quality evidence through high-quality clinical trials is time-consuming, thus limiting SSO_2_ therapy adoption and utilization in clinical practice at least for the next few years.

### 3.4 Future Directions

Future research should focus on addressing the existing gaps and refining therapeutic approaches to improve patient outcomes in treating AMI. To solidify the evidence base for SSO_2_ therapy, large-scale, multicenter, randomized controlled trials are needed. These trials should employ rigorous methodologies and include diverse patient populations to comprehensively evaluate the safety and efficacy of SSO_2_ in the real-world setting. Another crucial aspect is refining the patient selection criteria, which involves identifying the optimal timing for SSO_2_ therapy and characterizing the specific patient subgroups who would derive the greatest benefit. Factors such as infarct size, the extent of microvascular obstruction, and the time elapsed from symptom onset to reperfusion should be thoroughly investigated to guide personalized treatment decisions.

Long-term follow-up studies are essential to assess the sustained impact of SSO_2_ on critical clinical outcomes. These studies should evaluate the effects of SSO_2_ on mortality rates, the recurrence of ischemic events, the incidence of heart failure, and overall healthcare resource utilization. Additionally, developing comprehensive risk assessment models and predictive algorithms that incorporate various patient-specific factors, comorbidities, and hemodynamic status will aid in clinical decision-making and optimize the allocation of healthcare resources. Furthermore, alternative delivery methods for SSO_2_, such as microcatheter infusion or intravenous administration, should also be further explored. These approaches may help to minimize procedural complications and expand the accessibility of SSO_2_ therapy to a broader range of patients. Additionally, investigating the synergistic potential of SSO_2_ in combination with existing pharmacological therapies, including antithrombotic and cardioprotective agents, could lead to enhanced myocardial salvage and improved clinical outcomes.

Generating high-quality evidence that demonstrates the safety, efficacy, and clinical benefits of SSO_2_ therapy compared to existing standards of care is crucial for addressing reimbursement challenges. Developing interim guidelines and provisional reimbursement models based on promising early data could facilitate the initial adoption and utilization of SSO_2_ therapy in clinical practice. This would pave the way for broader acceptance as more comprehensive evidence becomes available.

Finally, continued efforts should focus on providing comprehensive training programs for interventional cardiologists and healthcare providers. These programs should emphasize the technical aspects of SSO_2_ therapy, its clinical benefits, and appropriate patient selection criteria. This will ensure the safe and effective implementation of SSO_2_ therapy in clinical practice. Furthermore, conducting detailed cost-benefit analyses and health economic studies will provide valuable insights into the financial implications of SSO_2_ therapy, supporting its wider adoption in healthcare systems.

## CONCLUSION

In conclusion, the emerging field of SSO_2_ holds significant promise in revolutionizing the management of acute myocardial infarction (AMI) and improving patient outcomes. It is evident that SSO_2_ therapy, administered post PCI within the critical timeframe of six hours from the onset of symptoms, offers substantial benefits in terms of reducing infarct size, preserving ventricular function, and preventing adverse left ventricular remodeling. However, despite the encouraging results observed thus far, several challenges remain, including the need for long-term follow-up studies to assess the impact of SSO_2_ therapy on mortality, recurrent ischemic events, and healthcare resource utilization. Furthermore, tailored therapeutic strategies based on patient-specific criteria, exploration of combination therapies, and optimization of the delivery methods are essential for successful integration of SSO_2_ therapy into routine clinical practice.

## AUTHORS' CONTRIBUTIONS

The authors confirm their contribution to the paper as follows: Conceptualization: AP; Visualization: MM; Writing - Original Draft Preparation: MRV, MB and NVA; draft manuscript: MB and NK. All authors reviewed the results and approved the final version of the manuscript.

## Figures and Tables

**Fig. (1):**
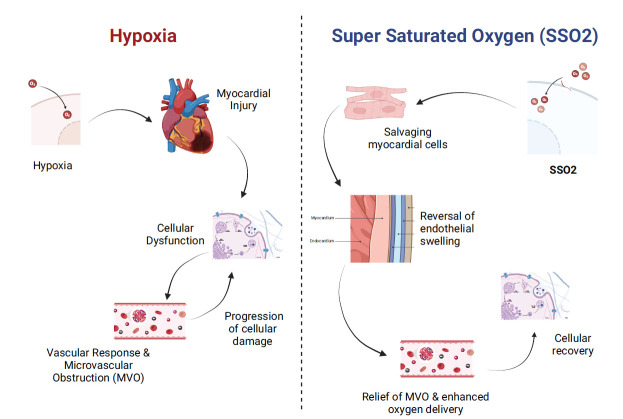
Impact of super saturated oxygen (SSO_2_) therapy on myocardial cell salvage and vascular recovery. This illustration demonstrates the therapeutic intervention of SSO_2_, showing its role in salvaging myocardial cells, reversing endothelial swelling, and improving oxygen delivery, leading to cellular recovery.

**Table 1:** Key findings of pre-clinical animal studies on SSO_2_.

**Study**	**Model Used**	**Intervention**	**Key Findings**
Spears *et al*. [16]	Canine	Intra-arterial aqueous oxygen infusion	▪ Preservation of LVEF in hypoxemic dogs *vs.* decline in LVEF in low-flow hypoxemic and normoxemic perfusion dogs
Spears *et al*. [16]	Canine	Intracoronary SSO_2_ post auto-reperfusion	▪ Increased mean arterial PaO_2_, improved LVEF, LV regional wall motion abnormalities, ST-segment deviation. ▪ Increased regional myocardial blood flow compared to normoxemic auto-reperfusion.
Spears *et al*. [17]	Porcine	Balloon occlusion followed by intracoronary SSO_2_	▪ Enhanced LVEF, smaller myocardial infarct size. ▪ Better preservation of myocardial structure, lower hemorrhage score. ▪ Reduced myeloperoxidase levels compared to control groups
Spears *et al*. [18]	Various animal models	SSO_2_ therapy post-MI	▪ Reduction in myocardial apoptosis, less endothelial cell edema, and preservation of endothelial nuclei, indicating improved structural integrity.
Kantor *et al*. [19]	Porcine	SSO_2_ infusion (PaO_2_ 622 mm Hg)	▪ Enhanced LV function and reduced infarct size, especially in pigs with baseline LVEF <5%.
Spears *et al*. [25]	Swine	Delayed SSO_2_ therapy post-reperfusion	▪ Significant improvements in infarct size and LVEF compared to control group.
Kaluza *et al*. [26]	Porcine	SSO_2_ post-primary PCI with stent implantation	▪ No coronary thrombi or adverse effects on coronary arteries or myocardium, trend towards reduced myocardial scarring, and improvement in LV function.
Kaluza *et al*. [27]	Swine	Chronic effects of SSO2 therapy post-MI	▪ No evidence of coronary thrombosis or myocardial or systemic toxicity from SSO_2_ therapy, trend towards reduced myocardial scarring, reduced infarct size, and improved global and regional contractility.

**Table 2:** Key findings of early clinical trials on SSO_2_.

**Study/Trial**	**Patient Population**	**Intervention**	**Summary of Key Findings**
AMIHOT I [14]	269 patients with acute STEMI	90-minute intracoronary SSO_2_ therapy	▪ Better regional wall motion score (-0.75 *vs.* -0.54; *P* = 0.03). ▪ Decreased area under ST-segment deviation time curve over 3 hours. ▪ No significant differences in major adverse cardiac events.
Dixon *et al*. [28]	29 patients with acute STEMI	Hyperoxemic Oxygen using TherOx^®^ device	▪ Improved echocardiographic regional wall motion score index. ▪ Increased mean LVEF at 24 hours (48.6% immediately after angioplasty *vs.* 51.8% at 24 hours). ▪ Safe and well-tolerated.
AMIHOT II [14]	301 patients with anterior STEMI	90-minute infusion of SSO_2_ therapy	▪ Smaller median infarct size (18.5% *vs.* 25% of LV mass) compared to control. ▪ Greater myocardial salvage in patients with baseline LVEF <40%.
IC-HOT [36]	100 anterior STEMI patients	60-minute infusion of SSO_2_ therapy	▪ Low net adverse cardiac event rate at 30 days (7.1%). ▪ Prevention of LV dilatation and remodeling.
Pilot Study [38]	20 patients with acute anterior STEMI	60-minute infusion of SSO_2_ therapy	▪ Feasible and safe delivery of SSO_2_ through a standard 5F diagnostic catheter in the LM. ▪ Mean myocardial salvage index of 50% at 3-5 days.

## LIST OF ABBREVIATIONS

AMIHOT I: Acute Myocardial Infarction with Hyperoxemic Therapy I
AMIHOT II: Acute Myocardial Infarction with Hyperoxemic Therapy II
AMI: Acute Myocardial Infarction
CMR: Cardiac Magnetic Resonance
FDA: Food and Drug Administration
HF: Heart Failure
IC-HOT: Evaluation of intracoronary hyperoxemic oxygen therapy in acute anterior myocardial infarction
INFUSE-AMI: INFusion of intracoronary Abciximab and Aspiration Thrombectomy in Patients with Anterior ST-segment Elevation Myocardial Infarction
IS: Infarct Size
ISO-SHOCK: Incorporating Supersaturated Oxygen in Shock
LAD: Left Anterior Descending
LM: Left Main
LV: Left Ventricular
LVEF: Left Ventricular Ejection Fraction
MI: Myocardial Infarction
MVO: Microvascular Obstruction
NACE: Net Adverse Clinical Events
PaO_2_: Partial Pressure of Arterial Oxygen
PCI: Percutaneous Coronary Intervention
SSO_2_: Supersaturated Oxygen
SPECT: Single Photon Emission Computed Tomography
STEMI: ST-elevated Myocardial Infarction
STEMI-CS: Acute Myocardial Infarction and Cardiogenic Shock
TIMI: Thrombolysis in Myocardial Infarction
TVR: Target Vessel Revascularization
